# Mixed Training Team Implementation of the Basic Emergency Care Course in Liberia

**DOI:** 10.5334/aogh.5477

**Published:** 2026-10-16

**Authors:** Nichole Michaeli, Gerald Ekwen, Katie Main, Sarah Anyango, Margi Massa Mabande, Frances Carter, Sterman Toussaint, Shada Rouhani

**Affiliations:** 1 University of Vermont Health, Department of Emergency Medicine, 111 Colchester Ave, Burlington, Vermont 05401, USA; 2 Partners In Health Liberia, S Baltimore Rd, Harper, Liberia; 3 MaineHealth Emergency Medicine, 22 Bramhall St, Portland, Maine 04102, USA; 4 J.J. Dossen Memorial Hospital, Maryland Ave, Harper, Liberia; 5 Partners In Health, 800 Boylston St Ste 300, Boston, Massachusetts 02199, USA; 6 Harvard Medical School, Department of Emergency Medicine, 75 Francis St, Boston, Massachusetts 02115, USA

**Keywords:** Basic Emergency Care, education, training, implementation

## Abstract

*Background:* The Basic Emergency Care (BEC) course was developed to improve the management of acute illness and injury in resource-limited settings. Although BEC materials are freely available, formal instructor credentialing requires time and resources that may limit rapid implementation. In remote areas, such as Maryland County, Liberia, innovative approaches to BEC training are needed.

*Objective:* To evaluate the effectiveness of a BEC course delivered by a mixed instructor team consisting of credentialed and non-credentialed facilitators in improving emergency care knowledge and self-efficacy among healthcare providers in Liberia.

*Methods:* We conducted a mixed-methods evaluation of two consecutive five-day BEC courses held in Maryland County, Liberia, in August 2022. Course participants included physicians, medical interns, physician assistants, and nurses. The instructional team included one credentialed BEC Registered Facilitator and four non-credentialed physician specialists. To promote course fidelity, instructors completed an independent review of course materials, participated in a two-day pre-course preparation workshop, and conducted daily planning and debriefing sessions throughout the training. The effectiveness of this model was evaluated by measuring improvements in participants’ emergency care knowledge and confidence using pre- and post-course examinations and self-efficacy surveys.

*Findings:* Participants demonstrated significant improvements in emergency care knowledge, with examination scores increasing from 64.0% pre-course to 86.6% post-course (*P* < 0.001). Self-efficacy surveys also showed substantial gains in participants’ comfort, confidence, and preparedness to manage critically ill and injured patients. Structured instructor preparation and ongoing coordination supported consistent course delivery despite the mixed facilitator model.

*Conclusions:* A BEC course delivered by a mixed team of credentialed and non-credentialed instructors significantly improved emergency care knowledge and self-efficacy among Liberian healthcare providers. This pragmatic, resource-conscious approach may offer a scalable strategy for expanding access to BEC training in remote settings while maintaining course quality and educational effectiveness.

## Introduction

Liberia’s 14-year civil war (1989–2003) devastated the country’s healthcare infrastructure; most health facilities were non-functional, much of the healthcare workforce had left, and many health professional institutions closed [1, 2]. Although the health sector experienced a gradual recovery in the following decade, the 2014–2015 Ebola virus disease outbreak pushed the fragile system into crisis [3, 4]. During the epidemic, 378 healthcare workers were infected with Ebola and 192 died [4]. In 2015, Liberia had just 0.3 physicians and 1.0 nurses and midwives per 10,000 people, which improved to 1.8 and 10.07 per 10,000, respectively, in 2022 [5, 6]. However, urban areas continue to attract most healthcare workers, while rural regions, such as Maryland County, struggle with severe shortages [7, 8].

In Maryland County, J.J. Dossen Memorial Hospital is the main government referral center, and Pleebo Health Center is the largest government primary health center [9, 10]. Partners In Health (PIH), an international non-governmental organization, has supported long-term health system strengthening at both facilities since 2015 [9, 10]. Emergency care and triage training was implemented for staff at both facilities from 2019 to 2020, which showed improvements in patient care [11]. However, high provider turnover, limited physician staffing, and increasing patient volumes at J.J. Dossen Memorial Hospital and Pleebo Health Center reflect the need for continued emergency care training.

The Basic Emergency Care (BEC) course was developed by the World Health Organization (WHO) in collaboration with the International Committee of the Red Cross (ICRC) and the International Federation for Emergency Medicine (IFEM) to train healthcare providers to manage acute illness and injury in resource-limited settings [12]. This open-access course utilizes lectures, small group discussions, and skills stations to teach all levels of healthcare providers to recognize, stabilize, and manage critically ill and injured patients. The BEC course has been successfully taught in over 80 countries, including Liberia [12–19].

Recent innovations in BEC delivery formats offer additional ways to access the information. The BEC self-paced course is now available online through the OpenWHO portal, and the BEC hybrid course can be accessed through the WHO Academy platform [20–21]. These formats can expand access to standardized content. However, these online courses are inaccessible in many remote or resource-limited areas, such as Maryland County, where internet connectivity is limited and implementation of new knowledge and skills requires hands-on support. In such contexts, in-person training remains essential.

Although the BEC course materials are open-source and free to access, the WHO and IFEM provide guidelines for recommended course instruction, and IFEM and regional societies, such as the African Federation of Emergency Medicine (AFEM), oversee the credentialing of trainers to ensure the fidelity of the course [22–24]. However, IFEM guidelines note that “organisations are welcome to deliver BEC training that falls outside of this certification framework,” and the WHO guidelines state that “a Ministry of Health may offer its own courses and provide certification through national or regional professional bodies” [22, 23]. However, the time and resources needed to formally credential BEC instructors are barriers to rapid implementation and distribution of critical emergency care knowledge and skills.

After evaluating available resources and time constraints, we elected to deliver BEC courses in Maryland County with a mixed instructor team consisting of both formally and informally trained BEC instructors. We report here the learning outcomes from this BEC course implemented with a mixed (credentialed and non-credentialed) instructor team.

## Methods

### Study design

We used a mixed-methods design to evaluate participant emergency care knowledge and confidence gains from BEC courses conducted by a mixed instructor team.

### Participants and study setting

Two consecutive five-day BEC courses were conducted in August 2022 in Maryland County, Liberia. Twenty-three participants, including general practitioner physicians, intern physicians, physician assistants, and nurses, were selected by the Ministry of Health (MOH) County Health Officer.

### Course instructors

Course instructors included one BEC Registered Facilitator (an emergency medicine physician) and four non-credentialed instructors (physicians specialized in emergency medicine, surgery, and obstetrics/gynecology), although one of the non-credentialed instructors had completed a formal BEC Training of Trainers (ToT) course [23]. All of the instructors reviewed the BEC course materials independently and as a team prior to teaching the BEC courses. The training team undertook several structured preparation strategies to maintain course fidelity and delivery, including a two-day pre-course preparation period. During the course, daily facilitator planning and debriefing meetings were conducted.

### BEC course

The five-day BEC courses included lectures, small group discussions, and skills stations covering the ABCDE approach, trauma, difficulty in breathing, shock, and altered mental status.

### Data collection and analysis

The effectiveness of the course implementation strategy was assessed with multiple-choice, pre- and post-course examinations and surveys, as well as a post-course feedback form. The pre- and post-course examinations were the standard versions developed as part of the BEC training package [24]. The pre- and post-course surveys were adapted from the BEC training package to improve clarity and relevance to our population and assessed participants’ confidence using a four-point Likert scale with responses ranging from 1 (strongly disagree) to 4 (strongly agree) and 1 (not confident at all) to 4 (very confident) [24]. Post-course feedback surveys assessed the participants’ satisfaction with the course using open-ended comments.

Mean scores from the pre- and post-course examinations were compared using the paired two-sample *t*-test, while qualitative data from the pre- and post-course surveys were compared using the exact binomial test for paired data. Statistical analyses were performed using SAS 9.4 (Cary, NC: SAS Institute Inc.). Qualitative data from the post-course feedback surveys were analyzed using thematic analysis.

### Ethics

This program and evaluation were conducted for quality improvement purposes and did not meet the criteria for human subject research.

## Results

### Demographics

A total of 23 healthcare workers from three healthcare facilities in Maryland County, Liberia participated in the course (Table 1). The participants consisted of four intern physicians, two general practitioner physicians, three physician assistants, and fourteen nurses. Fourteen participants worked at J.J. Dossen Hospital, eight at Pleebo Health Center, and one at Karloken/Edith Wallace Health Center. None of the participants had prior formal emergency medicine training or experience with the BEC course.

**Table 1:** Participant demographics.

WEEK	INTERN PHYSICIANS	GENERAL PRACTITIONER PHYSICIANS	PHYSICIAN ASSISTANTS	NURSES
Week 1	2	1	0	8
Week 2	2	1	3	6
**Total, *n* (%)**	**4 (17)**	**2 (9)**	**3 (13)**	**14 (61)**

### Pre- and post-course examination results

There was a significant improvement between pre-course examination scores and post-course examination scores for both weeks 1 and 2. Combined post-course examination scores (mean 86.6%, range 60–100%) showed a significant improvement (*P* < 0.001) compared to pre-course examination scores (mean 64%, range 44–80%) (Table 2).

**Table 2:** Pre- and post-course test scores.

WEEK	PRE-TEST (MEAN, RANGE)	POST-TEST (MEAN, RANGE)	*P*-VALUE
Week 1 (*n* = 11)	64.4% (44–80%)	87.6% (60–100%)	*P* < 0.001
Week 2 (*n* = 12)	63.7% (44–76%)	85.7% (72–100%)	*P* < 0.001
**Combined Week 1 and 2**	**64% (44–80%)**	**86.6% (60–100%)**	***P* < 0.001**

### Pre- and post-course survey results

The pre- and post-course surveys were completed by 23 participants. However, some participants did not answer all the questions. We excluded those participants from the pre- and post-survey pairing for those specific questions. Table 3 shows changes in self-reported confidence in providing emergency care and performing critical emergency skills.

**Table 3:** Pre- and post-course self-efficacy survey results.

QUESTION	PRE AND POST PAIRS (*N*)	PRE-COURSE (*N*, %)	POST-COURSE (*N*, %)	*P*-VALUE
**Participants who agree or strongly agree to the following (four-point Likert scale)**				
I feel comfortable handling any patient requiring EC.	22	16 (73%)	22 (100%)	***P* < 0.05**
I feel calm about seeing patients with emergencies	21	12 (57%)	20 (95%)	***P* < 0.05**
I feel that others in my clinical unit have the knowledge and skills to handle emergency care patients	22	13 (59%)	16 (73%)	*P* = 0.38
I feel that I have the skills to provide care in most emergencies	23	19 (83%)	23 (100%)	*P* = 0.13
I feel prepared to see emergency care patients in my clinical setting	23	19 (83%)	23 (100%)	*P* = 0.13
I feel confident seeing very ill patients	23	17 (74%)	21 (91%)	*P* = 0.22
I feel comfortable using WHO EC protocols	23	18 (78%)	23 (100%)	*P* = 0.06
I feel that I understand the ABCDE’s of BEC	23	21 (91%)	23 (100%)	*P* = 0.50
I feel that I have an organized approach that allows me to be prepared for all emergency care patients	23	12 (52%)	23 (100%)	***P* < 0.05**
I feel confident in my knowledge of EC	22	19 (86%)	22 (100%)	*P* = 0.25
I believe that EC trainings for generalist healthcare providers are important	23	23 (100%)	22 (96%)	*P* = 1.00
I believe that EC trainings for nurses are important	23	23 (100%)	22 (96%)	*P* = 1.00
**Participants who are confident or very confident in the following (four-point Likert scale)**				
Emergency Management of the acutely ill adult	18	13 (72%)	18 (100%)	*P* = 0.06
Emergency Management of the acutely ill child	18	9 (50%)	17 (94%)	***P* < 0.05**
Emergency Management of the injured adult	17	10 (59%)	17 (100%)	***P* < 0.05**
Emergency Management of the injured child	18	8 (44%)	17 (94%)	***P* < 0.05**
Emergency Management of the patient with Shock	18	13 (72%)	18 (100%)	*P* = 0.06
Emergency Management of the patient with AMS	18	7 (39%)	17 (94%)	***P* < 0.05**
Emergency Management of the patient with DIB	18	11 (61%)	18 (100%)	***P* < 0.05**
Understanding of emergency drugs	18	11 (61%)	18 (100%)	***P* < 0.05**
Managing an obstructed (blocked) airway	18	8 (44%)	18 (100%)	***P* < 0.05**
Providing oxygen to a patient with DIB	18	15 (83%)	18 (100%)	*P* = 0.25
Using an inhaler for a patient with DIB	18	15 (83%)	18 (100%)	*P* = 0.25
Managing a patient with bleeding problems	18	12 (67%)	18 (100%)	***P* < 0.05**
Immobilizing the spine in trauma patients	18	10 (56%)	18 (100%)	***P* < 0.05**

### Feedback surveys

The post-course feedback surveys were completed by all 23 participants. Analysis of participant feedback from all surveys revealed several recurring themes regarding the strengths and potential improvements of the course. Key themes included the perceived educational value of lectures, the effectiveness of hands-on skills sessions, expressions of gratitude toward facilitators, and recommendations for extending course duration or implementing periodic refresher training (Table 4).

**Table 4:** Results of feedback surveys.

THEME	EXAMPLE/QUOTE
Educational value	Participants felt lectures were educational and informative; skills sessions were highly appreciated, especially needle decompression and pelvic binding
Facilitator quality	*“I will like to take this time to appreciate all my facilitators for their knowledge and skills impacted in me. I have learned lots and promise to abide/put everything into practice.”*
Course duration	Several participants suggested increasing the time for practical sessions and extending the overall course duration.
Refresher training	Participants recommended refresher training courses at least annually for staff in the ED.

## Discussion

This program demonstrated the effectiveness of a BEC course taught by a credentialed BEC instructor and non-credentialed physician specialists to strengthen emergency medicine knowledge, skills, and confidence among Liberian healthcare workers. Pre- and post-course testing revealed a significant increase in knowledge, with mean scores improving from 64% to 86.6% (*P* < 0.001). In addition, self-efficacy surveys showed marked gains in participants’ comfort, confidence, and preparedness to manage emergency cases. The non-significant increase in self-assessed confidence on several survey questions likely reflects the small sample size and the ceiling effect given the participants’ high level of baseline confidence. These knowledge and confidence gains closely mirror those observed in courses conducted with certified instructors [14–19].

The results of these BEC courses clearly show the effectiveness of the facilitation team. While four of the facilitators were not formally credentialed BEC instructors, all had strong backgrounds in medical education and had previously taught emergency care and trauma principles. Their teaching experience, coupled with their dedication to independently learning the BEC material, enabled them to serve as effective instructors. The BEC Registered Facilitator shared prior expertise as a BEC instructor with the team and provided access to the BEC assessments, including the pre- and post-tests and surveys, which are not open-source. Utilizing this mixed instructor model helped to conserve resources while still improving participants’ emergency care knowledge, skills, and confidence. Without this approach, additional time and cost would have been required to formally train additional BEC facilitators or to engage external facilitators. While few BEC studies cite the cost of training, two studies indicate that the initial costs associated with certified BEC and ToT courses are approximately USD 5000, not including the cost of international faculty [25–26]. Expenses for international, certified facilitators have been reported as high as USD 4000 per course [26].

Our course delivery was strengthened through deliberate coordination and unified teaching approaches. A two-day pre-course preparation was held to review course content, standardize teaching approaches, and ensure alignment of practical skill demonstrations. Throughout the training week, daily facilitator planning and debriefing meetings were conducted to reflect on what went well, anticipate challenges, adjust the pacing of sessions, and coordinate upcoming activities. This structured approach promoted consistency across instructors, fostered collaborative problem-solving, and reinforced course fidelity, even with a mixed group of facilitators.

While WHO and IFEM credentialing protocols for BEC instructors are clearly defined [22, 23], there are no required ongoing assessments of clinical or teaching skills, meaning that facilitators may retain their credentials despite long periods without clinical practice or active teaching. This raises important questions about the assumption that initial certification alone ensures ongoing instructional quality. It may be worth considering whether credentialed facilitators should also incorporate formal pre-course review days to better ensure sustained competency and fidelity over time.

Despite the effectiveness of our instructors, there are important limitations to conducting a non-certified BEC course. Participants in this course will not be formally recognized as BEC Providers by IFEM or AFEM [23, 24]. Organizations seeking to implement this model must, therefore, weigh the benefits of faster, more cost-effective training against the benefits of formal credentialing. While this approach can expand access and conserve limited resources, it does not currently provide international recognition. However, these courses were conducted in partnership with the MOH and PIH, which provided a certificate that participants can use nationally as documentation of their knowledge and skills.

## Recommendations

Our experience shows that mixed instructor teams can effectively increase participants’ BEC knowledge and confidence. We propose this model as an alternative pathway to BEC instructor certification, in which a credentialed BEC instructor could provide oversight and ensure course fidelity by conducting pre-course content reviews, observing course delivery, providing daily structured feedback, and recommending non-credentialed instructors for full recognition based on teaching competence. This model could improve the expansion of BEC training in settings with limited access to credentialed BEC instructors. This approach balances the urgent need for timely, cost-effective training with the long-term benefits of formal credentialing. It also allows organizations to tailor implementation to their specific resources, timelines, and health system priorities. For Liberia and similar contexts, this pathway would offer a scalable solution for equipping healthcare providers with lifesaving skills, while building a team of BEC instructors necessary for sustained education. Future studies should rigorously evaluate this method of instruction.

## Conclusions

In our setting, a mixed facilitator team, composed of one BEC Registered Facilitator and four physicians with strong medical education backgrounds, effectively delivered BEC training. Participants showed significant improvements in both knowledge and self-efficacy, reporting increased confidence in managing critically ill and injured patients. This experience highlights a pragmatic, resource-conscious model that can expand access to standardized emergency care training in remote and underserved areas.
